# Thai Finger-Spelling Recognition Using a Cascaded Classifier Based on Histogram of Orientation Gradient Features

**DOI:** 10.1155/2017/9026375

**Published:** 2017-09-06

**Authors:** Kittasil Silanon

**Affiliations:** Department of Computer Engineering, Faculty of Engineering, Prince of Songkla University, Kathu, Phuket 83120, Thailand

## Abstract

Hand posture recognition is an essential module in applications such as human-computer interaction (HCI), games, and sign language systems, in which performance and robustness are the primary requirements. In this paper, we proposed automatic classification to recognize 21 hand postures that represent letters in Thai finger-spelling based on Histogram of Orientation Gradient (HOG) feature (which is applied with more focus on the information within certain region of the image rather than each single pixel) and Adaptive Boost (i.e., AdaBoost) learning technique to select the best weak classifier and to construct a strong classifier that consists of several weak classifiers to be cascaded in detection architecture. We collected 21 static hand posture images from 10 subjects for testing and training in Thai letters finger-spelling. The parameters for the training process have been adjusted in three experiments, false positive rates (FPR), true positive rates (TPR), and number of training stages (N), to achieve the most suitable training model for each hand posture. All cascaded classifiers are loaded into the system simultaneously to classify different hand postures. A correlation coefficient is computed to distinguish the hand postures that are similar. The system achieves approximately 78% accuracy on average on all classifier experiments.

## 1. Introduction

Sign language is a communication method for deaf or nonvocal people. For a sign language system, there are two main categories: (1) word-level vocabulary signs, which are signs of the hand shape, orientation and movement of the hands, arms, or body, and facial expressions simultaneously to represent word meanings, and (2) finger-spellings, which use only hand shape to spell the letters of the word in a spoken language, representing names, places, technical terms, and so on. However, most deaf and nonvocal persons, especially children, have problems with finger-spelling skills because finger-spelling is used infrequently in daily communication. Therefore, in order to help these people improve their skills, many systems specific to finger-spelling were proposed, for example, the American (ASL) (Dinh et al. [1], Feris et al. [2], Ricco and Tomasi [3], and Mo and Neumann [4]), British (BSL) (Goh and Holden [5]), Australian (Auslan) (Liwicki and Everingham [6]), Chinese (CSL) (Jiangqin and Wen [7] and Teng et al. [8]), and Japanese (JSL) (Fujimura and Liu [9] and Tabata and Kuroda [10]). In this work, we have focused on Thai finger-spelling (ThSL). Saengsri et al. [11] proposed a Thai letter finger-spelling by using the data glove, a motion tracker, and Neural Network theory to improve the accuracy of the system. Kanjanapatmata [12] presented an image recognition method for the Thai letter using a polar orientation histogram of the hand image and an artificial Neural Network. Sakulsujirapa et al. [13] presented an appearance feature lookup table to analyze hand posture patterns for identifying Thai letters in finger-spelling. Sriboonruang et al. [14] proposed a method combining the Zernike moment and wavelet moment to capture a hand's features and also using a fuzzy classification algorithm to classify Thai finger-spelling hand postures. Phitakwinai et al. [15] developed the Thai finger-spelling letters and words of the Thai sign language translation system using the scale-invariant feature transform (SIFT). However, they cannot achieve the critical criteria, such as accuracy, flexibility, and device constraints, and cannot run in real time.

**Figure 1:**
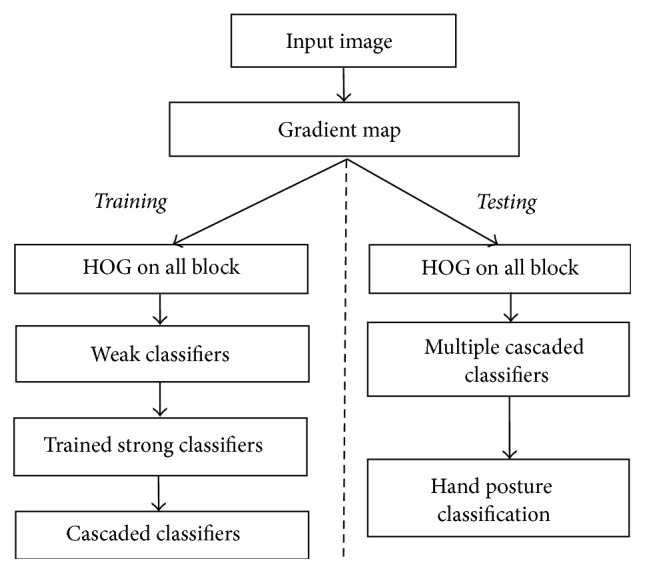
Thai letter finger-spelling recognition using HOG.

In this paper, we developed automatic classification to recognize 21 hand postures that represent letters in Thai finger-spelling. In our implementation, an object detection approach based on Histogram of Orientation Gradient (HOG) feature is applied as the main feature of hand postures which focuses more on the information within a certain region of the image rather than each single pixel. A feature is trained to be a weak classifier using a histogram comparison. In order to improve the detection speed, the weak classifiers are trained into strong classifiers by the AdaBoost algorithm, which were finally combined into a cascaded classifier for the detection procedure. The experiment is designed to adjust training parameters, false positive rates (FPR), true positive rates (TPR), and number of training stages (*N*), to achieve a suitable training model for 21 hand postures. All cascaded classifiers are loaded into the system simultaneously to classify different hand postures. The correlation coefficients are computed between inputs and pattern data to distinguish the hand postures that are similar. The system process in this method is shown in Figure 1.

## 2. Proposed Method

**Figure 2:**
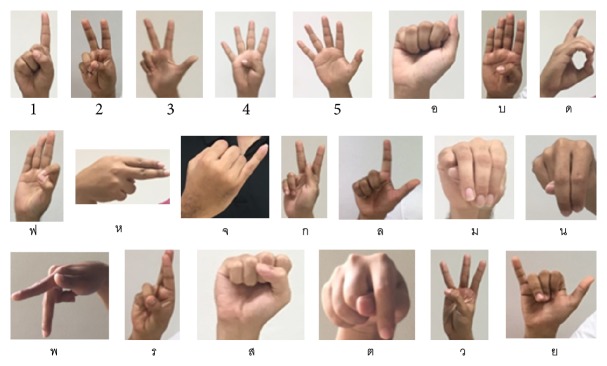
The 21 example hand postures.

This system recognizes 5 numbers and 16 letters of Thai finger-spelling (Silanon [17]) which use single hand postures. The digits and letters we recognize are the numbers “1,” “2,” “3,” “4,” and “5” and letters “
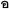
” (O ang), “
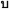
” (Bo bimai), “
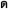
” (Do dek), “
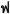
” (Fo fan), “
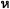
” (Ho hip), “
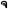
” (Cho chan), “
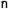
” (Ko kai), “
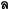
” (Lo ling), “
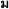
” (Mo ma), “
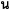
” (No nue), “
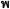
” (Po phan), “
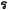
” (Ro ruea), “
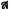
” (So suea), “
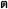
” (To tao), “
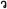
” (Wo waen), and “
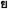
” (Yo yak). We collected these hand postures from 10 subjects who were asked to stand in front of white background. A web camera is used to capture an image resolution of 640 × 480 pixels in laboratory with a light condition. For each hand posture, there are 100 for training and 50 for testing. The 21 hand postures for Thai letter finger-spelling are shown in Figure 2.

Next we collected all hand postures into a dataset for a recognition process. We utilized HOG as the feature descriptor for each hand posture. Let us introduce HOG. The HOG features were used in many papers that address the object detection problem (Dalal and Triggs [18], Li et al. [19], Liu et al. [20], and Zhu et al. [21]). For HOG extraction, the first step of the calculation is the computation of the gradient values. This method requires filtering the gray scale image with the following filter kernels:

(1) Dx=−101,Dy=−101T.

Therefore, given an image I, we obtain the *x* and *y* derivatives using a convolution operation:

(2) Ixr,c=Ir,c+1−Ir,c−1,Iyr,c=Ir−1,c−Ir+1,c.

The orientation of the gradient is then transformed to polar coordinates (*θ*), with the magnitude of the gradient |*G*|:

(3) θ=tan−1⁡IyIx,G=Ix2+Iy2.

The image window is divided into a small spatial cell of size of 8 × 8 pixels. The cells are rectangular, and the histogram channels are spread over 0 to 180^0^, which determine 9 bins. We group the 2 × 2 cells into single* block feature* (**b**) and normalize the block feature to reduce the effect of change in contrast between images of the same object by its Euclidean norm:

(4) $$\begin{array}{c} b=\frac{b}{{\left\|b\right\|}^{2}+\varepsilon }. \end{array}$$

**Figure 3:**
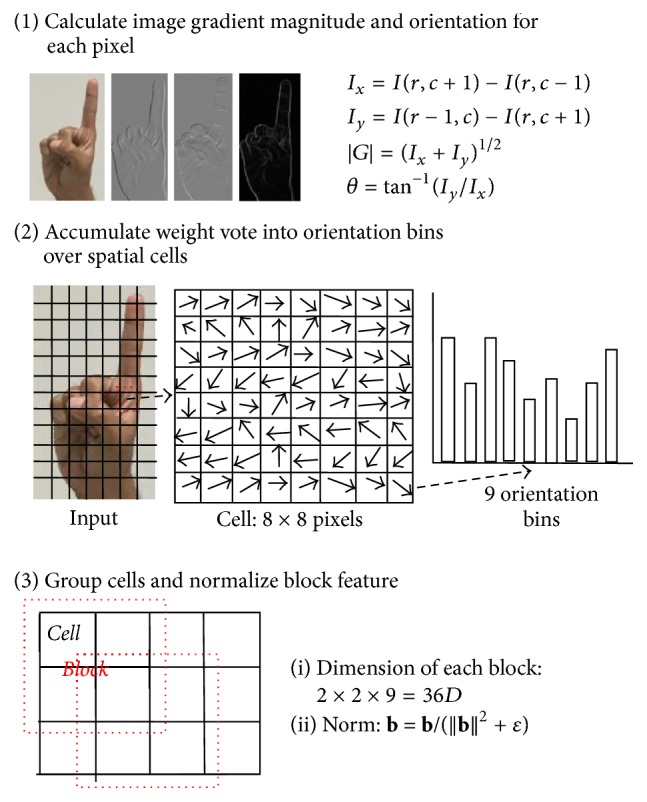
HOG feature calculation.

In this expression, *ε* is a small positive constant that prevents a division by zero. A dimension feature of each block is determined by the number of orientation bins in each cell. Therefore, there are 36 dimensions for a block feature.  Figure 3 showed the process of HOG feature calculations.

**Figure 4:**
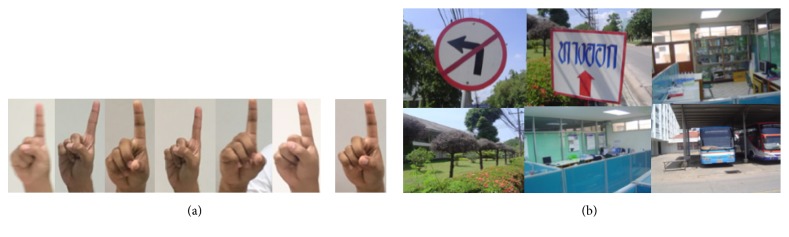
(a) Positive samples and (b) negative samples.

The second step is to study the construction of the weak classifier (*h*_*j*_) for hand postures. We estimate the distance between the histogram of an input feature (*x*_*j*_) and a model histogram *m*_*j*_. The model calculated the average histogram between all training positive examples. For each histogram of the feature set, we have its corresponding model *m*_*j*_. We define the weak classifier as a binary function *h*_*j*_(*x*):

(5) $$\begin{array}{c} h_{j}\left(\;x\;\right)=\left\{\begin{array}{cc} 1 & \text{if }d\left(x_{j},m_{j}\right)<\theta_{j}\\ 0 & \text{otherwise,} \end{array}\right. \end{array}$$

where *d*(*x*_*j*_, *m*_*j*_) is the histogram comparison (Negri et al. [22]) between the feature *x*_*j*_ and the model *m*_*j*_ and *θ*_*j*_ is the feature threshold. In practice, no single weak classifier can identify the object with high accuracy. We used the AdaBoost learning algorithm (Chen and Georganas [23], Pavani et al. [24], and Viola and Jones [25]), which can improve the accuracy detection. Now let us review the AdaBoost algorithm. The algorithm takes as input a set of *M* training samples, labeled as negatives (0) or positives (1): {(*x*_1_, *y*_1_) ⋯ (*x*_*N*_, *y*_*N*_)}, where *y*_*i*_ is the label of a certain instance *x*_*i*_, as shown in Figure 4.

Then a group of classifiers is tested from the set of samples and the best weak classifier, according to a minimum error, is chosen. Finally, the algorithm computes the parameter *α* associated with the chosen weak classifier, which measures the importance of the weak classifier's contribution to the final strong classifier. The process is repeated *T* times, extracting a new weak classifier per iteration. Thus the weak classifier and the corresponding weight are determined through the boosting procedure. The prediction of a strong classifier for the binary classification problem has the following form:

(6) $$\begin{array}{c} H\left(\;x\;\right)=\left\{\begin{array}{cc} 1,\text{object} & \overset{k}{\underset{i=1}{\sum }}\alpha_{i}h_{i}\left(x\right)\geq \frac{1}{2}\overset{k}{\underset{i=1}{\sum }}\alpha_{i}\\ 0,\text{clutter} & \text{otherwise.} \end{array}\right. \end{array}$$

The pseudocode of AdaBoost algorithm adapted to the object detection problem is shown in Pseudocode 1.

**Figure 5:**
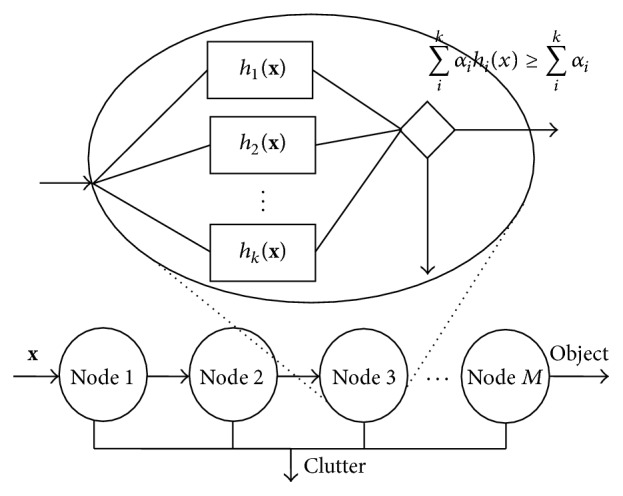
The structure of cascaded classifier.

For object detection, a cascaded classifier is built which consists of serially connected nodes labeling a test image as either object or clutter. Each node contains a boosted set of weak classifiers. In Figure 5, the third node of the cascaded classifier is expanded to show the *k* weak classifiers presented inside it. A given test image is scanned at all positions and scaled by the cascaded classifier. When an image subregion **x** is put to a node, it is classified by all the weak classifiers presented in the node, and the weighted average of their decisions is calculated as the final decision of that node. An image subregion is labeled as an object when it is identified as object by *M* nodes of the cascade (see Figure 5). On the other hand, if a subregion is labeled as a clutter by any node, it will not be processed by the successive nodes. Thus, a detection system based on cascaded classifier architecture will be fast in scanning the entire test image.

## 3. Experiments

The experiments are conducted in the laboratory with controlled light conditions. The positive training set images were collected as 100 original samples for each hand posture. However, we can also generate more positive samples from existing ones by varying the brightness or the contrast. Thus, we have a set of 500 training images for each hand posture. The negative samples come from 17,000 images without a hand posture. The cascaded training process involves two types of trade-offs (1) the number of stages (*N*) and (2) the threshold of true positive rate (TPR) and false positive rate (FPR) of each stage to achieve higher detection and a lower false positive rate. Unfortunately, finding this optimum is a tremendously difficult problem. In practice, a simple hypothesis structure is used to produce an effective classifier, which is highly efficient. Each stage in the cascade reduces the false positive rate and increases the true positive rate. A classifier is trained by adding a number of stages until the target for false positive rate and detection rate is met (these rates are determined by testing the detector on a testing set). A target is selected for the maximum reduction in false positive rate while maintaining the minimum decrease in detection.

**Figure 6:**
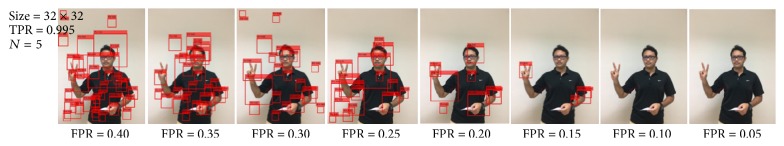
Result for “
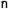
” (Ko kai) hand posture (adjusting FPR).

To test a hypothesis, there are three experiments based on testing different parameters to determine better performance of the classifier. We divide the experiment into three parts, that is, training with FPR, training with TPR, and training with *N*. In the first experiment, we tested the performance of correct classification with different FPR: the fraction of negative training samples incorrectly classified as positive samples or values in range (0,1]. This value is varied from 0.05 to 0.5 in step of 0.05. Other training parameters are fixed; for example, TPR is 0.995, *N* is 5, and training size is 32 × 32 pixels. To evaluate the performance of the training classifier, 50 images with a similar background and light condition (which are not used as training samples) for each hand posture class are tested. Each image has a resolution of 640 × 480 pixels.  Table 1 shows the performance of 21 trained classifiers for all test images.  Figure 6 shows some of the detection results of the “
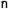
” (Ko kai) hand posture from each FPR value. From Table 1, outcomes are called “Hit” and “Miss” and “False” detection. The “Hit” detection is that hand posture is presented: the classification model must decide whether a hand posture is presented. The “Miss” detection is that hand posture is presented: the classification model decides otherwise. The “False” detection is an error in the evaluation process, in which a tested image is mistakenly found to be detected.

We heuristically find which value of FPR optimizes the performance of our classifier model. This value is varied experimentally based on each hand posture to achieve the best result. From experiment, the most suitable value of FPR for model is ranging from 0.05 to 0.30. The value of this parameter, for each hand posture class, is chosen from the case of maximum “Hit” detection (which is italicized bold in Table 1). For example, the hands posture class of “
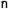
” (Ko kai) with a FPR of 0.15 is selected because it provides the maximum “Hit” detection result. By analyzing the experimental result carefully, we found that lower value for FPR can achieve less “False” detection. Nevertheless, results in “Miss” detection and “False” detection are still not suitable for use in real-time applications.

**Figure 7:**
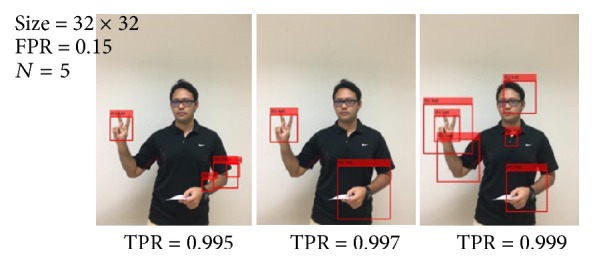
Result for “
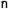
” (Ko kai) hand posture (adjusting TPR).

To reduce the probability of “Miss” and “False” detections, the second experiment is implemented to increase TPR: the fraction of correctly classified positive training samples or values in range (0,1]. This parameter was varied as 0.995 (1st experiment), 0.997, and 0.999, respectively. *N* is still 5. FPR for each hand posture class is also selected from the first experiment.  Table 2 shows the performance of 21 trained classifiers with different TPR. According to this experiment, the results of some hand posture classes have improved (which are italicized bold in Table 2). For instance, for the class of “
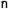
” (Ko kai), TPR of 0.997 is selected. However, this value does not affect to the “Miss” detection; but the “False” detection impact starts to occur as it has decreased slightly. A high value of the TPR results in a greater number of correct detections. However, it increases the training and detection times. The classification model is chosen from maximum “Hit” detection in each hand posture class. Although the classification model has improved, the “False” detection is still high.  Figure 7 shows some of detection results of the “
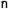
” (Ko kai) hand posture from each TPR value. To reduce the number of “False” detections, the third experiment is implemented by increasing the number of *N*. The 5th, 6th, 7th, 8th, 9th, and 10th stages were trained. FPR and TPR are selected from the first and the second experiments.  Table 3 shows the results of training stage variation.

**Figure 8:**
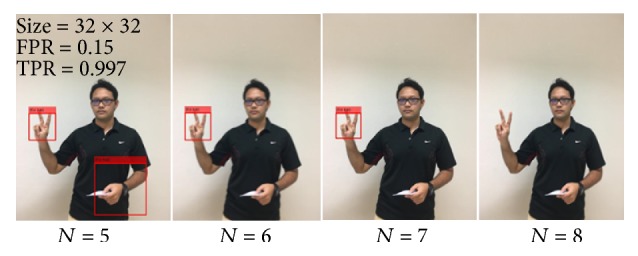
Result for “
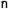
” (Ko kai) hand posture (adjusting *N*).

In most cases, classifiers with more stages achieved lower “False” detection. At the same time, classifiers with more stages provided more result in the “Miss” detection category as well. Classifiers with many states can have an overfitting model problem. An overfitting model generally occurs when a model is excessively complex, such as having too many training cycles or parameters relative to the number of observations. The model begins to memorize training data rather than learning to generalize from trend. Therefore, its performance is good on the training examples, while the performance on unseen data becomes worse. There is no reliable method to select the classification that always works. Therefore, a target of the classification model is selected for the maximum reduction in “False” detection and minimum decrease in “Hit” detection (which are italicized bold in Table 3).  Figure 8 shows some of the detection results of “
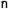
” (Ko kai) hand posture from each number of stages.

After the target classification models have been selected, we implement a multiple cascades structure to classify different hand postures. In structure, all cascades are loaded into the system simultaneously, and each cascade is responsible for detecting a single hand posture. Rectangle detection for different labels is used to tell which hand posture is detected. Based on the experimental results, we found that this method is fast enough to run in real time when we load all trained cascaded classifiers at the same time. Confusion may occur between hand postures. For example, the “
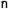
” (Ko Kai) hand posture and number “2” hand posture may be confused with each other. However, this confusion can be resolved by computing the correlation coefficient between the detection results, with a set of appropriate reference images of the hand postures. Then, we pick the matched hand posture by choosing the one that gives the maximum correlation coefficient. We computed the correlation coefficient (*r*) as follows:

(7) $$\begin{array}{c} r=\frac{\sum_{m}\sum_{n}\left(A_{mn}-\bar{A}\right)\left(B_{mn}-\bar{B}\right)}{\sqrt{\sum_{m}\sum_{n}{\left(A_{mn}-\bar{A}\right)}^{2}\sum_{m}\sum_{n}{\left(B_{mn}-\bar{B}\right)}^{2}}}. \end{array}$$

**Figure 9:**
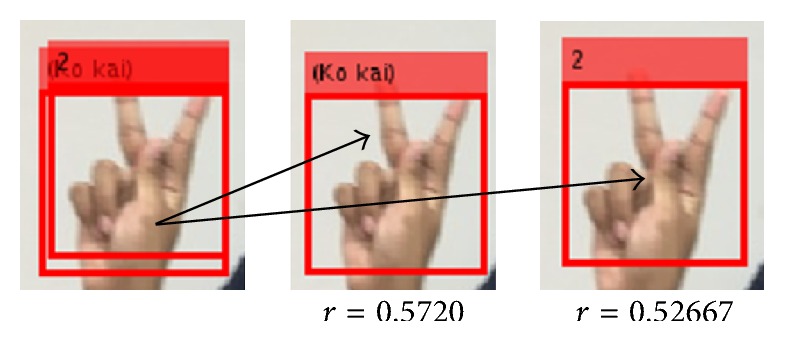
Correlation coefficient.

Here *A* and *B* are images of the same size. $\bar{A}$ and $\bar{B}$ are the means of image elements. An example of the correlation coefficient of the “
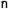
” (Ko kai) hand posture and number “2” hand posture is shown in Figure 9, with correlation coefficients of 0.5720 and 0.5267, respectively.

**Figure 10:**
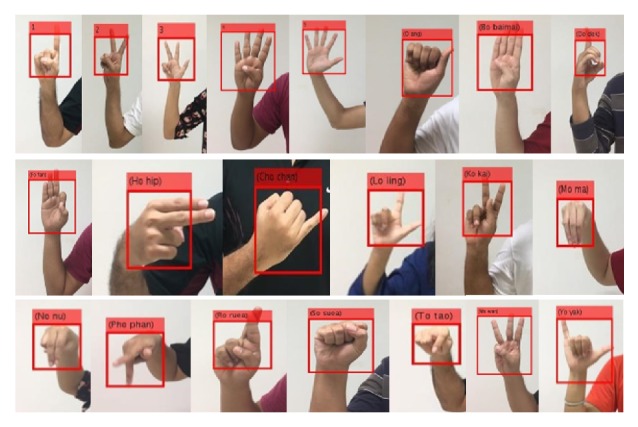
All hand postures detection.

Table 4 gives the confusion matrix for the detection of all hand posture classes, using a combination of all cascades, which were tested with 50 test images for each hand posture class. Rows are targets and sum up to one and columns are predictions. This shows confusion between similar-looking hand postures such as “
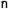
” (Ko kai) confused with “2” (6%) and also “
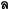
” (Lo ling), “
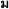
” (Mo ma), and “
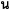
” (No nu). By analyzing the detection results, we found that some of the “Miss” detections are caused by the excessive in-plane or out-of-plane rotations due to hand posture variations and finger-spelling styles of different users. For the “False” detection, we found that the classification error might have occurred because there are some hand postures in Thai finger-spelling which are similar. For example, hand postures of “
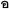
” (O ang), “
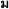
” (Mo ma), and “
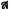
” (So suea) are all represented by a closed fist but differ only in the thumb position (depending on subject's dexterity), leading to higher confusion levels. Besides, the majority of “False” detections happened in small image areas. However, these small false detection boxes can be easily eliminated by defining a threshold for rectangular size. All hand postures detection is shown in Figure 10.

**Figure 11:**
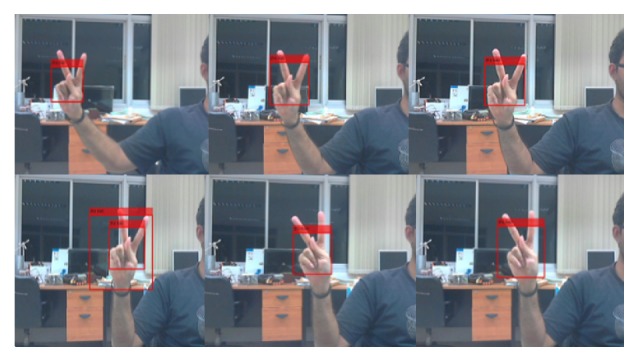
Real-time detection for the “
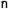
” (Ko kai) hand posture.

To give general comparison between previous methods and our proposed method, some existing research works involving Thai finger-spelling recognition are shown in Table 5. We compared the general conditions not only for our method but also for some previous research that used other additional devices such as a sensor glove or a color glove. Regarding the background of the image, some researchers set background to a constant color. In terms of the outfit, users are asked to wear long-sleeves shirts. Concerning the number of the letters that can be recognized in the system, our system is not as good as the method that used additional devices such as glove based method because the image is not as good as a signal from an electronic sensor, especially when fingers occlude or stick together. For methods that use only camera images, it is also hard to compare recognition performance achieved from different datasets for testing in Thai finger-spelling. For the recognition rate, our average classification precision is around 78% for 21 hand postures classification. Although our work does not yield a more significant result compared to other techniques, by analyzing other conditions (see Table 5), they need to set background such as black or white color, additional device is required such as color glove to separate hand from other parts of body, and most of existing works cannot run in real time, while our system does not need to do any preprocessing or segmentation before computing the finger-spelling recognition and is fast enough to be run in real-time situation and clutter background as shown in Figure 11.

## 4. Conclusion

We proposed an approach to recognize hand posture in real time with a single web camera as the input device. The approach focused on hand posture recognition with Histogram of Orientation Gradient (HOG) and the AdaBoost learning algorithm. The Histogram of Orientation Gradient feature can effectively describe the hand posture pattern with a computation of gradient orientation. This feature allows the system to be able to distinguish the hand postures that are similar. The AdaBoost learning algorithm can greatly speed up detection performance and construct a strong classifier by combining a sequence of weak classifiers. The experimental results were tested by adjusting training parameters, false positive rates (FPR), true positive rates (TPR), and number of training stages (*N*), to achieve the best classifier. A target of the classification model is selected for the maximum reduction in false detection and minimum decrease in detection. Based on the cascaded classifier, a multiple cascaded structure was implemented to classify different hand postures. The correlation coefficient must be computed when hand postures confuse each other. From experimental result, we found that the average classification accuracy is around 78%. For work comparison, our method does not need to do any preprocessing or segmentation before computing the finger-spelling recognition and is fast enough to be run in real time.. Furthermore, this method can be used with other problems in object detection field such as human, car, or symbol detector. In future work, we will implement the sequence recognition for other letters in Thai finger-spelling. Some letters in Thai finger-spelling occur from combination of hand posture. For example, “
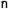
” (Ko kai) combined with digit “1.” This sequence of hand posture will be translated to “
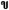
” (Kho Khai). For sequence recognition, a Finite State Machine (FSM) or a Hidden Markov Model (HMM) can be used to define the rule for the recognition process. Furthermore, if information of finger was taken into account and trained with more dataset images, then the errors of the classifier should be reduced.

## Figures and Tables

**Pseudocode 1:**
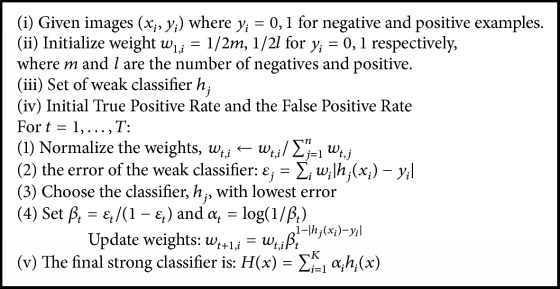
The AdaBoost algorithm.

**Table 1:** The performance of 21 trained classifiers (adjusting FPR).

Class	FPR = 0.05			FPR = 0.10			FPR = 0.15			FPR = 0.20			FPR = 0.25			FPR = 0.30		
	Hit	Miss	False	Hit	Miss	False	Hit	Miss	False	Hit	Miss	False	Hit	Miss	False	Hit	Miss	False
“1”	16	34	0	22	28	6	33	17	42	39	11	99	42	8	177	***45***	***5***	***198***
“2”	41	9	1	40	10	12	44	6	55	***49***	***1***	***138***	8	2	279	48	2	379
“3”	43	7	3	47	3	12	***49***	***1***	***58***	48	2	81	48	2	117	43	7	669
“4”	24	26	0	40	10	17	***41***	***9***	***51***	37	13	89	40	10	229	31	19	520
“5”	30	20	2	45	5	17	46	4	47	***48***	***2***	***91***	44	6	158	30	20	410
“ 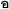 ”	47	3	0	49	1	6	50	0	20	***50***	***0***	***69***	46	4	131	46	4	158
“ 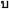 ”	5	45	0	28	22	8	21	29	80	33	17	140	***42***	***8***	***276***	37	13	376
“ 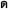 ”	29	21	4	39	11	8	41	9	22	***48***	***2***	***42***	42	8	194	32	18	234
“ 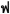 ”	30	20	1	29	21	4	40	10	81	***44***	***6***	***164***	27	23	231	30	20	316
“ 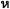 ”	6	44	1	22	28	9	15	35	35	18	32	95	23	27	244	***33***	***17***	***376***
“ 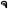 ”	39	11	0	42	8	32	***44***	***6***	***114***	36	14	203	31	19	227	26	24	502
“ 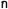 ”	36	14	0	44	6	3	***47***	***3***	***34***	45	5	220	38	12	633	40	10	899
“ 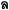 ”	***47***	***3***	***0***	44	6	0	45	5	36	43	7	112	42	8	198	43	7	354
“ 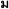 ”	25	25	3	32	18	29	36	14	78	38	12	133	***40***	***10***	***276***	36	14	366
“ 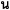 ”	24	26	2	33	17	16	39	11	92	42	8	99	***45***	***5***	***301***	36	14	293
“ 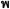 ”	36	14	8	36	14	14	39	11	90	***46***	***4***	***153***	39	11	293	39	11	570
“ 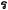 ”	18	32	1	38	12	6	39	11	21	***44***	***6***	***54***	26	24	647	6	24	786
“ 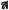 ”	12	38	1759	47	3	8	***49***	***1***	***6***	49	1	87	47	3	107	41	9	483
“ 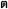 ”	36	14	0	43	7	11	42	8	19	***47***	***3***	***66***	44	6	102	44	6	234
“ 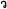 ”	16	34	0	23	27	12	30	20	35	39	11	68	35	15	363	***40***	***10***	***278***
“ 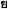 ”	42	8	0	43	7	11	42	8	39	***46***	***4***	***39***	39	11	113	43	7	242

**Table 2:** The performance of 21 trained classifiers (adjusting TPR).

	TPR = 0.995 (1st experiment)			TPR = 0.997			TPR = 0.999		
	Hit	Miss	False	Hit	Miss	False	Hit	Miss	False
“1”	45	5	198	45	5	305	***46***	***4***	***247***
“2”	49	1	138	***49***	***1***	***129***	48	2	102
“3”	49	1	58	49	1	59	***49***	***1***	***32***
“4”	41	9	51	32	18	16	***41***	***9***	***24***
“5”	48	2	91	45	5	49	47	3	62
“ 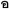 ”	50	0	69	49	1	87	49	1	77
“ 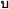 ”	42	8	276	28	22	295	35	15	197
“ 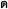 ”	48	2	42	43	7	86	43	7	69
“ 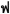 ”	44	6	164	41	9	124	37	13	98
“ 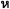 ”	33	17	376	22	28	351	29	21	361
“ 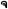 ”	44	6	114	44	6	118	***45***	***5***	***95***
“ 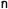 ”	47	3	34	***47***	***3***	***21***	46	4	79
“ 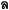 ”	47	3	0	46	4	0	43	7	1
“ 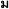 ”	40	10	276	***43***	***7***	***194***	40	10	218
“ 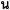 ”	45	5	301	***45***	***5***	***159***	37	13	418
“ 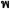 ”	46	4	153	46	4	178	41	9	167
“ 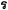 ”	44	6	54	***46***	***4***	***83***	44	6	60
“ 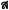 ”	49	1	6	***50***	***0***	***22***	50	0	29
“ 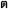 ”	47	3	66	45	5	65	***47***	***3***	***42***
“ 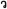 ”	40	10	278	36	14	469	30	20	467
“ 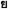 ”	46	4	39	46	4	73	44	6	107

**Table 3:** The performance of 21 trained classifiers (adjusting *N*).

Class	*N* = 5 (2nd experiment)			*N* = 6			*N* = 7			*N* = 8			*N* = 9			*N* = 10		
	Hit	Miss	False	Hit	Miss	False	Hit	Miss	False	Hit	Miss	False	Hit	Miss	False	Hit	Miss	False
“1”	46	4	247	41	9	301	***44***	***6***	***127***	38	12	44	20	30	7	9	21	5
“2”	49	1	129	***48***	***2***	***32***	45	5	9	45	5	2	21	29	0	31	19	0
“3”	49	1	32	***48***	***2***	***8***	44	6	5	44	6	3	45	5	3	43	7	5
“4”	***41***	***9***	***24***	33	17	14	27	23	7	25	25	0	18	32	3	12	38	0
“5”	48	2	91	***46***	***4***	***29***	42	8	8	36	14	5	32	18	1	35	15	1
“ 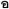 ”	50	0	69	50	0	41	50	0	8	***50***	***0***	***7***	49	1	1	49	1	1
“ 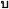 ”	42	8	276	***31***	***19***	***99***	13	37	29	7	43	9	3	47	2	2	48	0
“ 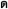 ”	48	2	42	42	8	13	***43***	***7***	***8***	37	13	6	43	7	0	36	14	1
“ 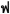 ”	44	6	164	41	9	28	***40***	***10***	***12***	30	20	2	31	19	0	22	28	1
“ 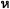 ”	***33***	***17***	***376***	19	31	141	21	29	37	11	39	7	7	43	10	9	41	4
“ 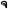 ”	45	5	95	40	10	50	***44***	***6***	***7***	40	10	2	36	14	0	37	13	2
“ 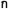 ”	***47***	***3***	***21***	39	11	8	41	9	0	24	26	0	31	19	0	26	24	0
“ 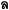 ”	47	3	0	44	6	0	42	8	0	***47***	***3***	***0***	41	9	0	45	5	0
“ 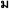 ”	43	7	194	42	8	148	***44***	***6***	***76***	34	16	14	23	27	8	20	30	9
“ 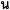 ”	45	5	159	43	7	69	***42***	***8***	***46***	33	17	6	31	19	12	24	26	0
“ 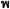 ”	46	4	153	44	6	102	***42***	***8***	***26***	36	14	11	35	15	0	36	14	1
“ 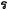 ”	46	4	83	***48***	***2***	***52***	41	9	10	36	14	3	22	28	2	18	32	1
“ 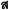 ”	50	0	22	***49***	***1***	***16***	42	8	0	43	7	1	36	14	1	34	16	1
“ 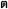 ”	47	3	42	***44***	***6***	***18***	42	8	4	33	17	2	34	16	0	26	24	0
“ 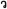 ”	40	10	278	40	10	252	38	12	141	***38***	***12***	***17***	16	34	7	11	39	3
“ 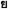 ”	46	4	39	44	6	65	***44***	***6***	***11***	42	8	0	41	9	0	39	11	1

**Table 4:** Confusion matrix of all hand posture detection.

	“1”	“2”	“3”	“4”	“5”	“ 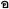 ”	“ 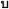 ”	“ 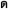 ”	“ 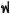 ”	“ 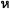 ”	“ 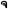 ”	“ 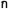 ”	“ 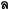 ”	“ 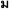 ”	“ 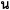 ”	“ 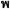 ”	“ 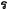 ”	“ 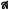 ”	“ 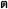 ”	“ 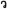 ”	“ 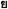 ”	miss
“1”	***0.78***	0	0	0	0	0.06	0	0	0	0	0	0	0	0	0.1	0	0.06	0	0	0	0	0
“2”	0.02	***0.82***	0.02	0	0	0	0	0	0	0	0.02	0	0	0.04	0	0	0	0	0	0	0	0.08
“3”	0	0.06	***0.84***	0	0	0	0	0	0	0	0	0.02	0.04	0	0	0	0	0	0	0	0	0.04
“4”	0	0	0	***0.78***	0.08	0	0	0	0	0	0	0	0	0	0.02	0	0	0	0	0.06	0	0.06
“5”	0	0	0	0.08	***0.84***	0	0	0	0	0	0	0	0	0	0	0	0	0	0	0.04	0	0.04
“ 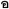 ”	0.06	0	0	0	0	***0.8***	0	0	0	0	0	0	0.02	0	0	0	0	0.06	0	0	0.06	0
“ 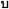 ”	0	0	0	0	0	0	***0.78***	0	0.06	0	0	0	0	0.02	0	0	0	0.06	0.02	0.02	0	0.04
“ 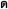 ”	0.02	0	0	0	0	0	0.02	***0.8***	0.06	0	0	0	0	0.02	0	0	0	0.02	0	0	0	0.06
“ 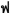 ”	0	0	0	0	0	0.06	0.1	0	***0.74***	0	0	0	0	0	0	0	0	0	0	0.02	0	0.08
“ 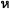 ”	0.06	0	0	0	0	0.02	0	0	0.02	***0.76***	0.04	0	0	0	0	0	0	0.02	0	0.02	0	0.06
“ 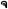 ”	0	0	0	0	0	0.08	0	0	0.04	0	***0.74***	0	0	0.04	0	0	0	0	0	0	0	0.1
“ 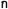 ”	0	0.06	0	0	0	0	0	0	0	0	0	***0.84***	0.02	0.02	0.02	0	0	0	0	0	0	0.04
“ 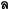 ”	0	0	0.04	0	0	0.02	0.04	0.02	0	0	0	0.06	***0.8***	0	0	0	0	0	0	0	0.02	0
“ 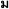 ”	0	0	0	0	0	0.02	0	0.04	0	0	0	0	0	***0.72***	0.04	0	0	0	0.02	0.02	0	0.14
“ 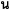 ”	0	0	0	0	0	0.04	0	0.02	0	0	0	0	0	0.08	***0.7***	0	0	0	0.1	0	0	0.06
“ 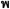 ”	0.04	0	0	0	0	0	0.04	0.02	0.02	0	0	0.02	0	0	0	***0.74***	0	0.02	0.02	0	0	0.08
“ 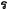 ”	0.04	0	0	0	0	0.04	0	0.02	0	0	0	0	0.06	0.02	0	0	***0.78***	0	0	0	0	0.04
“ 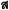 ”	0.04	0	0	0	0	0.04	0	0	0	0	0	0	0	0.04	0	0	0.04	***0.8***	0	0	0	0.04
“ 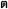 ”	0	0	0	0	0.02	0.02	0	0	0.02	0	0	0	0	0.06	0.08	0	0	0	***0.72***	0	0	0.08
“ 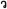 ”	0	0	0	0.12	0.04	0	0	0	0	0	0	0	0	0	0	0	0	0	0	***0.78***	0	0.06
“ 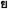 ”	0	0	0	0	0	0.08	0	0	0	0	0	0	0	0	0.04	0.02	0	0	0	0	***0.82***	0.04

**Table 5:** General comparison with existing systems.

Work	Device	Background	Outfit	Real time	Letters	Recognition rate
Saengsri et al. [11]	Sensor glove	No	No	Yes	16	94.44%
Kanjanapatmata [12]	No	Yes	Yes	No	15	72%
Veerasakulthong [16]	Color glove	Yes	Yes	No	31	88.26%
Sriboonruang et al. [14]	No	Yes	Yes	No	N/A^*∗*^	72%
Sakulsujirapa et al. [13]	No	Yes	Yes	No	42	81.43%
Phitakwinai et al. [15]	No	Yes	Yes	No	15	79.90%
Our method	No	No	No	Yes	21	78%

^*∗*^N/A: not available.
